# Evaluation of the Moral Distress Intensity and Its Relationship with the Quality of Work Life among Nurses Working in Oncology Wards in Shiraz, Southwest of Iran

**DOI:** 10.1155/2022/7977039

**Published:** 2022-10-25

**Authors:** Zahra Molazem, Leila Bagheri, Majid Najafi Kalyani

**Affiliations:** ^1^Community Based Nursing and Midwifery Research Center, School of Nursing and Midwifery, Shiraz University of Medical Sciences, Shiraz, Iran; ^2^Student Research Committee, School of Nursing and Midwifery, Shiraz University of Medical Sciences, Shiraz, Iran; ^3^School of Nursing and Midwifery, Shiraz University of Medical Sciences, Shiraz, Iran

## Abstract

**Background:**

Moral distress defined as an incident that prevents the appropriate functioning of individuals in spite of having the required knowledge. Nurses are commonly exposed to moral distress while doing their professional roles, which can influence the quality of their work life. The present study aimed to assess the relationship between the moral distress intensity and work life quality amongst nurses.

**Methods:**

In this descriptive-correlational study, 180 nurses working in oncology wards were selected through census based on inclusion criteria (associate or higher degrees, having at least six months of clinical work experience, and not suffering from anxiety disorders). The data were collected using Corley's questionnaire (2001) and Brooks' investigation of work life quality of nurses (2001). Then, the data were entered into the SPSS 22 software and were analyzed using the Independent Sample *t*-test, One-way ANOVA, and Pearson's correlation coefficient.

**Results:**

The nurses' mean scores of moral distress and work life quality were 136.63 ± 27.39 and 133.83 ± 25.40, respectively. The results revealed a negative significant relationship between the nurses' moral distress and work life quality (*P* < 0.001) (*r* = −0.53). There was also a significant difference between the nurses' sex and work life quality (*P* < 0.05).

**Conclusion:**

Identifying the moral distress intensity and work life quality of nurses and proper planning from the authorities can improve job satisfaction in nurses and resulting to higher quality of care.

## 1. Introduction

Due to being encountered with patients and spending time beside their bed more than other healthcare providers, nurses are prone to ethical issues and constantly face ethical decisions [1]. One of the ethical issues in nursing is moral distress, which has been defined as an incident that prevents the appropriate functioning of individuals in spite of having the required knowledge [2, 3]. Nurses encounter moral distress in situations where they know the proper practice, but are unable to do so due to stress [4]. A review of the literature showed low to moderate and high levels of moral distress among nurses [5]. For many years, moral distress has been known as a problem in nurses' performances [6]. Corley reported a moderate intensity and repetition of moral distress in nurses. It was also stated that the ethical atmosphere in the workplace was effective in moral distress and approximately 25% of nurses resigned from their job because of moral distress [3]. In Iran, Tajalli et al. found a high level of moral distress among nurses [7]. Furthermore, a moderate level of moral distress was found among Iranian nurses in the studies conducted by Ghasemi et al. [8] and Mohammadi et al. [9]. Ramos et al. in a study on Brazilian nurses found a moderate level of moral distress [10].

Moral distress can result from different factors including the lack of manpower in clinical settings, inadequate quality of care provided by physicians and nurses, negligence and medical errors, and incompetence of colleagues [11]. Besides, most nurses' moral distress can be attributed to dying patients, unnecessary tests, inadequate and incomplete treatment by colleagues, pain and resentment caused by invasive diagnostic and treatment methods, and treatment of patients to meet the needs of the organization [12]. These conditions occur due to the situations of patients and their families as well as the conflicting demands of patients and their families from their treatment teams [11]. Constant moral distress can lead to such problems as discouragement from professional life, reduced job satisfaction, burnout, dislike, decreased job retention, job change, leaving the profession, and minimum interaction with patients and their families, eventually exacerbating the shortage of nurses [12].

Inability to provide compassionate care due to moral distress may also have detrimental effects on the quality of care [5]. Therefore, attention to nurses' situations has been highly regarded by managers in order to increase the efficiency of hospitals [13]. In this context, a set of real working conditions in organizations such as salaries and benefits, welfare facilities, health and safety considerations, participation in decision-making, management methods, and diversity and richness of jobs, called quality of work life, should be taken into account [14]. Evidence has indicated that the quality of work life can be influenced by two factors, namely demographic and environmental variables [14]. Factors affecting dissatisfaction with the quality of work life include lack of income and fair rewards, safe and healthy working conditions, opportunities for the use and development of human capacity, and professional growth [15]. In a study on nurses working in Iranian hospitals, the quality of work life was low and the most important factors were inadequate and unfair payment, organization's failure to solve staff's problems, poor managerial support, job insecurity, high job pressure, promotion policies, and unfair and insufficient participation in decision-making [16]. Another study by Austin et al. showed that moral distress affected the quality of work life amongst physicians and nurses [17]. The important point to note is that nurses in challenging wards such as Intensive Care Units (ICUs) and oncology care, in addition to high functional skills, consider the psychological support of patients and their families, which is often associated with ethical challenges for them [18]. A prior research revealed a higher level of moral distress in nurses caring for cancer patients compared to others [19].

As working in the field of oncology, nursing is challenging and many factors causes moral distress considering its direct and indirect effects on nurses' quality of work life, and lack of similar studies on the issue, the present study aims to assess the relationship between the moral distress intensity and work life quality amongst oncology nurses.

## 2. Materials and Methods

### 2.1. Study Design

In this descriptive-correlational study, 180 nurses working in oncology wards of the hospitals affiliated to Shiraz University of Medical Sciences were selected via census in 2020. They were selected in the form of census because of the small number of nurses working in oncology wards and limited area of the study.

### 2.2. Recruitment

The inclusion criteria of the study were having associate or higher degrees in the field of nursing, having at least six months of clinical work experience as a nurse in the oncology ward, and not suffering from anxiety disorders (self-report). The exclusion criterion was unwillingness to continue cooperation in the study and incomplete completion of questionnaires. After getting the approval from the Ethics Committee of Shiraz University of Medical Sciences, the introduction letter was taken by the researcher to the hospitals where the study was conducted. With the coordination and permission of hospitals managers, the researcher visited the nurses in different shifts (morning, evening, and night) and distributed the questionnaire among them. The completed questionnaires were collected in the next shifts.

### 2.3. Data Collection

The study data were collected using a demographic information form, Corley's Moral Distress Questionnaire, and Brooks' Quality of Work Life Questionnaire. The demographic information form included age, sex, marital status, education level, type of employment, type of shiftwork, work experience, history of leaving the service, and history of anxiety disorders. This form was designed after reviewing the literature and using experts' opinions.

#### 2.3.1. Corley's Moral Distress Questionnaire

This questionnaire was designed by Corley in 1995 and was revised in 2001. It contained 36 questions in three areas of ignoring the patient, decision-making ability, and professional-functional competence. The items could be responded through a seven-point Likert scale, with six representing the highest level of moral distress and zero showing the absence of moral distress [20]. Thus, the minimum and maximum scores of the questionnaire were 0 and 216, respectively. Accordingly, scores 0-72, 73-144, and 145-216 indicated low, moderate, and severe moral distress, respectively. The validity and reliability of this questionnaire were measured by Corley, revealing Cronbach′s alpha = 0.90 [3]. This questionnaire was also evaluated by Motevallian et al. in Iran [21]. Its reliability was also measured by Beikrmoradi et al., indicating Cronbach′s alpha = 0.93, and its validity was approved [22].

#### 2.3.2. Brooks Quality of Work Questionnaire (2001)

This questionnaire consisted of 42 questions divided into four dimensions as follows: (1). work and family life (questions 1-7), (2). work plan (questions 7-18), (3). field of work (questions 17-37), and (4). work atmosphere (questions 38-42). The items could be responded through a six-point Likert scale (strongly disagree = 1, disagree = 2, relatively disagree = 3, relatively agree = 4, agree = 5, and strongly agree = 6). Thus, the total score of the questionnaire could range from 42 to 252, with higher scores representing a higher quality of work life. Additionally, the lowest and highest scores of work and family life, work plan, field of work, and work atmosphere subscales were 7 and 42, 10 and 60, 20 and 120, and 5 and 30, respectively [23]. The validity and reliability of this questionnaire were measured by Brooks, revealing Cronbach′s alpha = 0.90 [24]. In Iran also, Khani et al. assessed the validity and reliability of this questionnaire and reported Cronbach′s alpha = 0.93 [25].

### 2.4. Ethical Considerations

After obtaining approval from the Ethics Committee of Shiraz University of Medical Sciences (Ethics code: IR.SUMS.REC.1397.706) and getting introduction letter to enter hospitals for making the necessary arrangements, the objectives of the research were Explained to the participants. Informed written consent was obtained and they were also assured about the confidentiality of their information. Additionally, they were informed about responding to the questionnaires as well as the voluntary nature of the study.

### 2.5. Data Analysis

The data were entered into the SPSS 22 software and descriptive and analytical statistics were used according to the study objectives. Data analysis was done via Independent Sample *t*-test, One-way ANOVA, and Pearson's correlation coefficient. *P* < 0.05 was considered statistically significant.

## 3. Results

This study was conducted on 180 nurses working in oncology wards. Approximately half of the nurses (48.9%) aged below 30 years. The majority of the nurses were female and 83.9% of them had Bachelor's degrees. In addition, 53.30% of the nurses were married and the rest were single. Besides, 88.9% of the nurses worked in rotational shifts and 42.2% were formal workers (Table 1).

The mean score of moral distress was 136.63 ± 27.39. Additionally, the nurses' mean score of quality of work life was 133.83 ± 25.40 (Table 2).

The results showed that there was no significant difference between the nurses' demographic characteristics and moral distress. Both male and female nurses had moderate levels of moral distress (Table 3).

Work life quality was significantly higher in female nurses than in males (*P* < 0.05). However, there was no significant difference between the nurses' work life quality and other demographic features (Table 4).

The results of Pearson's correlation test demonstrated that the higher the nurses' moral distress, the lower their quality of work life would be (*r* = −0.53, *P* < 0.001) (Table 5). There was also a significant relationship between the dimensions of work life quality and moral distress (*P* < 0.001).

## 4. Discussion

This study aimed to assess the relationship between the moral distress intensity and work life quality amongst nurses working in oncology wards of the hospitals affiliated to Shiraz University of Medical Sciences in 2020.

The study results indicated that moral distress was moderately high in the nurses working in oncology wards. Borhani et al. [12], Mahdavi et al. [2], and Dodek et al. [26] also reported a high level of moral distress among nurses. Nurses are more prone to moral distress compared to other healthcare team members [1]. Ritchie et al. [27] and Prentice et al. [28] reported high levels of moral distress among nurses in ICUs and neonatal units, respectively. However, contradictory results were achieved in two Iranian studies. Behbodi et al. [29] showed a noticeably low intensity of moral distress in the majority of pediatric nurses. This could be attributed to the type of nurses' activities; the aforementioned study was performed on pediatric nurses, while the current one was done on the nurses working in oncology units. Nonetheles, Fard et al. revealed a high intensity of moral distress in the majority of nurses working in pediatric wards [30]. This difference could result from the study design. This study was conducted in private and in state hospitals, with a larger sample size in all clinical wards.

The results of the present study showed no significant difference between the nurses' moral distress and demographic characteristics. These results were in line with those of some other investigations. For instance, the results of the research by Mardani et al. showed that moral distress was not significantly related to shiftwork and marital status among nurses [31]. However, this study was not limited to oncology nurses and was done on nurses in all units. Sirilla et al. also revealed no significant link between nurses' age and work history and moral distress [32]. Similarly, Behbodi et al. reported that except for age, variables like marital status, number of children, and educational level were not associated with moral distress [29]. In contrast, Mahdavi et al. disclosed that nurses' demographic characteristics could affect their moral distress [2]. Additionally, Behbodi et al. found that the intensity of moral distress is decreased by age [29]. Furthermore, Fard et al. indicated that nurses working in ICUs had a higher level of moral distress, which was directly related to their experiences [30].

In the studies performed by Suleiman et al. [33], Alharbi et al. [34], and Kaddourah et al. [35] on Saudi Arabian nurses, a moderate work life quality was observed. In Iranian studies also, a medium quality of life was found among nurses. Similarly, Labani et al. [36], Javanmardnejad et al. [37], Shafipour et al. [38], and Mohammadi et al. [39] showed a moderate quality of life among most nurses.

On the contrary, Shabaninejad et al. [40] and Dehghan Nayeri et al. [41] indicated that nurses had low to medium levels of work life quality. Raeissi et al. [16] also showed a low quality of work life in nurses working in Iranian hospitals. Nonetheless, some research projects revealed a high work life quality amongst nurses. For example, Kumar et al. demonstrated that nurses' work life quality and job satisfaction were relatively favorable in India [42].

In the present study, the work life quality was higher in female nurses in comparison with males. In the same line, Lebni et al. [36], Moradi et al. [43], and Raeissi et al. [16] disclosed that female nurses' work life quality was more desirable compared to males. The low work life quality of male nurses might be associated with their participation in stressful nursing activities, which could negatively affect their perceived work life [44]. These results were in agreement with those obtained by Suleiman et al. in 2019 regarding marital status, but not age [33]. In general, people become more skilled as they age, which is effective in promoting their work life quality. Mahdavi et al.[2] and Borhani et al. [12] stated that nurses' work life quality had nothing to do with their demographic characteristics. Consistently, Mohammadi et al. indicated that nurses' work life quality was not associated with their university degrees [45]. In the research by Jafari et al. also, nurses' work life quality varied in terms of their demographic characteristics [46]. Moreover, Moradi et al. showed that experienced nurses had a better work life quality compared with others [43]. According to Alharbi et al., higher work experience was effective in higher work life quality [34]. In the study by Lebni et al., work life quality was significantly related to participants' characteristics [36].

The current study findings revealed a decrease in the nurses' quality of work life with increase in moral distress. Austin et al. showed that moral distress in nurses significantly impacts on professional quality of life [17]. In a study by Mahdavi et al., moral distress was accompanied by many disadvantages that had negative effects on nurses' profession [2]. Consistent with the results of the present study, Borhani et al. also reported a significant correlation between moral distress and professional stress [12]. Moreover, Rushton et al. found a correlation between moral distress and burn out of nurses in high acuity care settings [47]. Joolaee et al. reported a significant relationship between moral distress and job satisfaction. They conclude that job satisfaction as a component of quality of work life can be affected by moral distress [48]. Moral distress is an important issue in nursing and may affect nurses' decision making in daily of work life [49]. As moral distress can affect on the quality of care and quality of life, morally distressed nurses are prone to burn out [50, 51].

One of the potential limitations of this study was its small sample size. Additionally, some nurses could not be accompanied due to their job status and work pressure, which might have affected their responses.

The most important innovation aspect of this study was conducting among oncology nurses for the first time in Iran. According to the organizational conditions governing Iran's hospitals in terms of lack of support and the increasing number of cancer patients, the level of moral distress is increasing, which has not yet been measured in terms of the quality of work life among oncology nurses.

## 5. Conclusion

The results of the present study showed a negative correlation between moral distress and quality of work life among nurses working in oncology wards. Making arrangements for counseling, expressing morally stressful situations, and teaching strategies for coping with moral distress can be helpful. Since moral distress is a multidimensional phenomenon affected by various environmental, occupational, organizational, and personal factors, the hospital management system is required to pay attention to this issue to reduce nurses' moral distress. It is necessary to pay attention to this phenomenon and hold training classes. Furthermore, ethics committees in hospitals are suggested to improve nurses' work environments. Improving the quality of work life can, in turn, lead to satisfaction, reduced moral distress, and improved patient care.

## Figures and Tables

**Table 1:** Demographic characteristics of participants.

Variable		Frequency	Percent
Age	20-30	88	48.9
	31-40	77	42.8
	>40	15	8.3
Sex	Male	56	31.1
	Female	124	68.9
Marital status	Single	79	43.9
	Married	96	53.3
	Divorced	5	2.8
Level of education	Associate	17	9.4
	Bachelor	151	83.9
	Master	12	6.7
Type of employment	Formal	76	42.2
	Semiformal	35	19.4
	Contractual	12	6.7
	Corporate	18	10.0
	Passing the compulsory medical service program	39	21.7
Type of shift	Fixed	20	11.1
	Rotational	160	88.9
History of work leave	Yes	21	11.7
	No	159	88.3

**Table 2:** The mean scores of moral distress and work life quality and its dimensions in nurses.

Variable		Mean ± SD
Moral distress		136.63 ± 27.39
Quality of work life		133.83 ± 25.40
	Work life–family	18.52 ± 5.81
	Work plan	32.07 ± 6.48
	Work field	68.29 ± 14.69
	Work atmosphere	14.96 ± 3.39

**Table 3:** The results of evaluation of the nurses' moral distress according to the demographic characteristics.

Demographic characteristics		Mean	SD	*P* value	Test
Age (years)	<30	139.06	27.35	0.090	One-way ANOVA
	31 - 40	131.96	27.30		
	>41	146.33	25.24		
Gender	Female	136.52	26.47	0.97	*t*-test
	Male	136.68	27.90		
Level of education	Associate	140.23	24.94	0.58	One-way ANOVA
	Bachelor	135.72	28.40		
	Master	142.83	15.06		
Marital status	Single	137.57	25.64	0.43	One-way ANOVA
	Married	136.66	28.99		
	Divorced	121.20	21.86		
History of work leave	Yes	137.52	27.74	0.87	*t*-test
	No	136.51	27.43		
Type of shift	Fixed	147.00	25.18	0.07	*t*-test
	Rotational	135.33	27.45		
Type of employment	Formal	136.72	27.12	0.30	One-way ANOVA
	Semiformal	135.51	25.84		
	Contractual	123.33	34.65		
	Corporate	134.33	29.45		
	Passing the compulsory medical service program	142.59	25.56		

**Table 4:** The relationship between the nurses' work life quality and demographic variables.

Demographic characteristics		Mean	SD	*P* value	Test
Age (years)	<30	133.48	25.93	0.277	One-way ANOVA
	31 - 40	136.04	25.81		
	>41	124.60	18.27		
Gender	Male	127.04	24.82	0.015	*t*-test
	Female	136.90	25.16		
Level of education	Associate	124.70	26.92	0.29	One-way ANOVA
	Bachelor	134.74	25.00		
	Master	135.33	27.81		
Marital status	Single	133.56	27.61	0.93	One-way ANOVA
	Married	133.85	23.77		
	Divorced	137.80	23.78		
History of work leave	Yes	128.86	23.97	0.34	*t*-test
	No	134.49	25.58		
Type of shift	Fixed	131.10	17.47	0.61	*t*-test
	Rotational	134.18	26.25		
Type of employment	Formal	134.16	23.62	0.93	One-way ANOVA
	Semiforma	133.03	23.04		
	Contractual	135.83	33.44		
	Corporate	137.67	26.16		
	Passing the compulsory medical service program	131.54	28.61		

**Table 5:** The correlation between moral distress and work life quality among the nurses.

	Moral distress	*P* value
	Correlation coefficient (*R*)	
Work life quality	-0.53	<0.001
Work–family life	-0.41	<0.001
Work program	-0.47	<0.001
Work field	-0.45	<0.001
Work atmosphere	-0.36	<0.001

## Data Availability

The datasets used and analyzed during the current study are available from the corresponding author on reasonable request.
